# Supplementary material for: Analysis of key parameters influencing the permeability of cement sheath based on multiphysical fields
Source: PLoS One. 2025 Jan 16;20(1):e0315934. doi: 10.1371/journal.pone.0315934 (PMC11737677; doi:10.1371/journal.pone.0315934)
Supplement: Attachment — Submitted filename: renamed_a2a8f.pdf [file pone.0315934.s002.pdf]

Reviewer's comments on manuscript entitled

"*Analysis of key parameters influencing the permeability of cement sheath based on multiphysical fields*" authored by Luo Weia *et al* (PONE-D-24-39005)

This reviewer carefully read the manuscript and found it to be well-written. The study focused on the finite element methods to develop a simulation model for cement sheath seepage. It analyzes parameters influencing seepage flow, cement sheath length, permeability, gap structure, pressure difference, and fluid characteristics. However, before accepting the manuscript, the corresponding author/s should make some minor corrections pointed out in the manuscript, including typo errors.

Also, in some parts of the Results and Discussion section, sufficient discussion with recently published citations is not done; only results are described.
