# Supplementary material for: Analysis of key parameters influencing the permeability of cement sheath based on multiphysical fields
Source: PLoS One. 2025 Jan 16;20(1):e0315934. doi: 10.1371/journal.pone.0315934 (PMC11737677; doi:10.1371/journal.pone.0315934)
Supplement: Attachment — Submitted filename: Respond to reviewer.doc [file pone.0315934.s003.doc]

**Reviewer #1: Report**

**Title: Analysis of key parameters influencing the permeability of cement sheath based on multiphysical fields**

**This paper investigates the seepage characteristics of cement sheaths, considering the flow properties of porous media, by utilising finite element analysis in Comsol Multiphysics software. The model described in the present paper seems to have some novelty in the area of fluid flow through porous media (cement sheaths), which may be applicable in diagnostic testing for any oil or gas well with cement sheath pressure. The manuscript is written well and seems suitable for publication in the esteemed journal, but it requires further minor revision to enhance its overall quality.**

**The following comments must be addressed before making a decision for this manuscript:**

**1. After the precise review of the paper, it is found that the basic introduction of cement sheath and other keywords are missing. Please incorporate it in the introduction section.**

Dear reviewer

We has been added the basic introduction of cement sheath and other keywords.

The cement sheath is a crucial component in oil and gas well construction, filling the annular space between the borehole and casing. Its primary functions are to seal the wellbore, preventing fluids such as oil, gas, and water from migrating along the outside of the casing, protecting the reservoir and surrounding environment, and providing structural support to the wellbore. During drilling and completion operations, cement slurry is injected into the annular space between the borehole and casing, where it hardens to form a cement sheath. This sheath needs to exhibit good mechanical strength, adhesion, and low permeability to effectively withstand various factors like external pressure changes, temperature fluctuations, and chemical corrosion. Additionally, the cement sheath must maintain stable sealing performance throughout the well’s lifespan to prevent any fluid leakage. This stability is vital for protecting the subsurface reservoirs and ensuring long-term well productivity. In petroleum engineering, the permeability of the cement sheath is critically important, as it directly affects the sealing effectiveness of the well. If the cement sheath's permeability increases, fluids may penetrate through it, potentially leading to fluid migration between the reservoir and surrounding formations. This can result in contamination between formations and could even harm the surrounding ecological environment.[1-2].

**2. Improve the typos errors in whole manuscript. For now:**

**“CO2” in line 75; “..” in line 182; “(3.9 \times 10{-9})” in line 190; Check line 206;**

**“1.54×10-2” in line 279; “from 1 mm to 1.19×10-1 m3/d” in line 417.**

Dear reviewer

I sincerely apologize for our oversight. We have carefully corrected these errors and conducted a thorough review of the entire paper.

**3. There are many abbreviations used in this paper. It is advised to include a table for all abbreviations used in the paper.**

Dear reviewer

There are only two abbreviations for the full text, we think it is not necessary to establish abbreviations, and please reconsider.

sustained casing pressure (SCP)

casing-cement interface (CCI)

**4. It is also advised to provide citations for all governing equations and utilised formulas. Also, use proper syntax (comma or full stop) at the end of displayed equations**

Dear reviewer

We have added references and added proper syntax.

**5. Give the references for the chosen numerical range of parameter values of emerging parameters.**

Dear reviewer

We have given the references for the chosen numerical range of parameter values of emerging parameters.

**6. Try to exclude access use of indent in each paragraph.**

Dear reviewer

We have reduced paragraph inentation.

**7. For instance, address the following articles in context of flow through porous media:**

**a) https://doi.org/10.1016/j.chaos.2024.114961**

**b) https://doi.org/10.1002/zamm.202200047**

**c) https://doi.org/10.1016/j.chaos.2024.114726**

**d) https://doi.org/10.1016/j.cjph.2024.01.017**

**b) https://doi.org/10.1016/j.cjph.2024.03.046**

Dear reviewer

We have added references to the following article.

a) https://doi.org/10.1016/j.chaos.2024.114961

28.Yadav P K, Yadav N. Impact of heat and mass transfer on the magnetohydrodynamic two-phase flow of couple stress fluids through a porous walled curved channel using Homotopy Analysis Method[J]. Chaos, Solitons & Fractals, 2024, 183: 114961.

b) https://doi.org/10.1002/zamm.202200047

29.Yadav P K, Verma A K. Analysis of two non‐miscible electrically conducting micropolar fluid flow through an inclined porous channel: Influence of magnetic field[J]. ZAMM‐Journal of Applied Mathematics and Mechanics/Zeitschrift für Angewandte Mathematik und Mechanik, 2023, 103(1): e202200047.

**Reviewer #2: This reviewer carefully read the manuscript and found it to be well-written. The study focused on the finite element methods to develop a simulation model for cement sheath seepage. It analyzes parameters influencing seepage flow, cement sheath length, permeability, gap structure, pressure difference, and fluid characteristics. However, before accepting the manuscript, the corresponding author/s should make some minor corrections pointed out in the manuscript, including typo errors.**

Dear reviewer

We have conducted a thorough review of the entire text, including checks for grammatical errors and typos.
